# Persistent urban heat

**DOI:** 10.1126/sciadv.adj7398

**Published:** 2024-04-10

**Authors:** Dan Li, Linying Wang, Weilin Liao, Ting Sun, Gabriel Katul, Elie Bou-Zeid, Björn Maronga

**Affiliations:** ^1^ Department of Earth and Environment, Boston University, Boston, MA, USA.; ^2^ Department of Mechanical Engineering, Boston University, Boston, MA, USA.; ^3^ Guangdong Key Laboratory for Urbanization and Geo-simulation, School of Geography and Planning, Sun Yat-sen University, Guangzhou, China.; ^4^ Institute for Risk and Disaster Reduction, University College London, London, UK.; ^5^ Department of Civil and Environmental Engineering, Duke University, Durham, NC, USA.; ^6^ Department of Civil and Environmental Engineering, Princeton University, Princeton, NJ, USA.; ^7^ Institute of Meteorology and Climatology, Leibniz University Hannover, Hannover, Germany.; ^8^ Geophysical Institute, University of Bergen, Bergen, Norway.

## Abstract

Urban surface and near-surface air temperatures are known to be often higher than their rural counterparts, a phenomenon now labeled as the urban heat island effect. However, whether the elevated urban temperatures are more persistent than rural temperatures at timescales commensurate to heat waves has not been addressed despite its importance for human health. Combining numerical simulations by a global climate model with a surface energy balance theory, it is demonstrated here that urban surface and near-surface air temperatures are significantly more persistent than their rural counterparts in cities dominated by impervious materials with large thermal inertia. Further use of these materials will result in even stronger urban temperature persistence, especially for tropical cities. The present findings help pinpoint mitigation strategies that can simultaneously ameliorate the larger magnitude and stronger persistence of urban temperatures.

## Teaser

The thermal memory of urban materials prolongs extreme temperature hazards in cities.

## INTRODUCTION

Urbanization is arguably one of the most profound human-induced land cover changes (*1*, *2*), and its imprints on the climate system are a subject of inquiry and debate (*3*–*5*). Most cities experience the urban heat island (UHI) effect with higher temperatures recorded compared to surrounding rural areas. Much attention has been dedicated to the magnitude and diurnal/seasonal variations of UHIs (*6*). However, the difference between urban and rural land in terms of temperature persistence (i.e., the tendency for temperature anomalies to continue for an extended period) remains unexplored. Moreover, a mechanistic link between how urbanization alters surface properties and the persistence of temperatures in cities continues to be elusive.

Addressing these research gaps at timescales of heat waves (i.e., multiday extreme temperature events) is of particular importance. Heat is one of the most important drivers of weather-related mortality (*7*, *8*). Studies have reported that heat waves are associated with mortality rates that exceed the anticipated impacts from single hot days (*9*, *10*). Every 1-day increase in heat wave duration is found to be associated with an increase of mortality risk by 0.38% in the United States, and such increases of mortality risk are even higher (2.50%) in the northeastern United States (*11*). Understanding how urban surface characteristics modify the temperature persistence is thus a prerequisite for quantifying health risks associated with urban heat waves and developing interventions to reduce heat-related illnesses in cities.

## RESULTS

### Quantifying temperature persistence in a global climate model

**Fig. 1.**
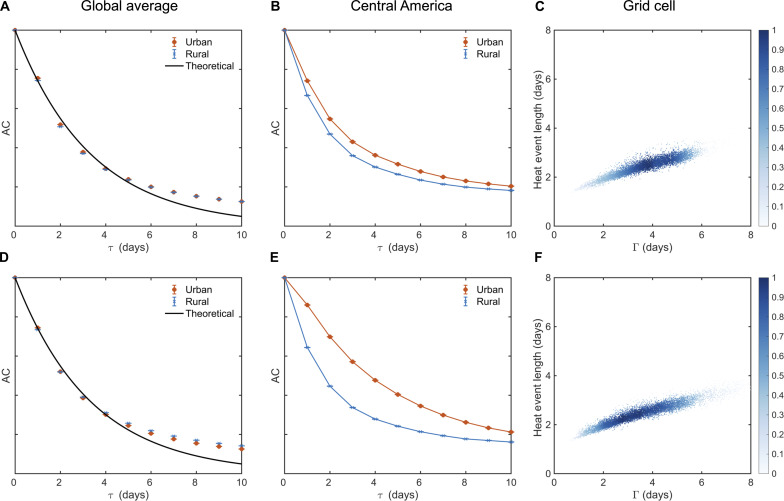
Daily urban and rural near-surface air and surface temperature anomalies exhibiting red-noise behavior. (**A** to **C**) Near-surface air temperature. (**D** to **F**) Surface temperature. (A and D) The global average temporal autocorrelation (AC) as a function of lag (τ) for urban (red) and rural (blue) temperature anomalies. The average is performed over all grid cells that have urban fractions larger than 0.1% (4241 in total). The black line is the theoretical result for a red-noise process (an exponential decay with a decay rate of 1/Γ). (B and E) Similar to (A) and (D) but for averages over Central America (latitude: 7°N to 18°N, longitude: 82°W to 118°W). (C and F) Relation between Γ and the average length of heat events, which are defined as periods when the daily temperature anomaly is positive and larger than 1 SD during the study period (1991–2010). Data in (C) and (F) include both urban and rural results and are for all grid cells that have urban fractions larger than 0.1%. The color in (C) and (F) indicates data density. The upper and lower bounds in (A), (B), (D), and (E) represent the 95% confidence intervals for the spatial mean AC values. The 95% confidence intervals are small because of the very large sample size.

The persistence of daily temperature can be quantified using autocorrelation and spectral analyses (Materials and Methods). In this study, we use the near-surface air temperature and radiative surface temperature (hereafter referred to as surface temperature) simulated by the Community Earth System Model (CESM) during the period of 1991–2010 (Materials and Methods). Before applying autocorrelation and spectral analyses, the long-term linear trend and the mean annual cycle of temperature are removed (see Materials and Methods), yielding daily temperature anomalies. Figure 1 (A and D) features the globally averaged temporal autocorrelation for near-surface air temperature and surface temperature, respectively. Results suggest that both urban and rural near-surface air temperature and surface temperature anomalies have signatures of a “red-noise” process (i.e., the autocorrelation decays exponentially), consistent with Hasselmann’s stochastic climate model results (*12*, *13*) and the meteorological literature (*14*, *15*). The spectral results are shown in fig. S1, where signatures of a red-noise process are again observed: The spectra of both near-surface air temperature and surface temperature approach *f*^−2^ (*f* indicates frequency) at high frequencies. The computed autocorrelation and spectra are broadly consistent with results from prior studies (*16*, *17*), which nonetheless did not examine the urban-rural contrasts.

For a red-noise process, the decay rate of the temporal autocorrelation (Fig. 1, A and D) and the peak of the premultiplied spectra (fig. S1, A and B) can be used to define a characteristic timescale (Γ) that quantifies temperature persistence (see Materials and Methods). In the following, the timescale (Γ) characterizing the decay rate of the temporal autocorrelation, which is estimated from lag-1 autocorrelation (see Materials and Methods), will be used (called the persistence timescale hereafter).

The global averaging of urban and rural results (Fig. 1, A and D) leads to small urban-rural differences that may indicate similar temperature persistence between urban and rural land. However, this finding does not imply that urban and rural persistence timescales are similar at regional and local scales. Figure 1 (B and E) shows the results for Central America where the urban autocorrelation decays slower than the rural autocorrelation, implying stronger urban temperature persistence. The reason for the stronger urban temperature persistence in this region will be discussed later.

**Fig. 2.**
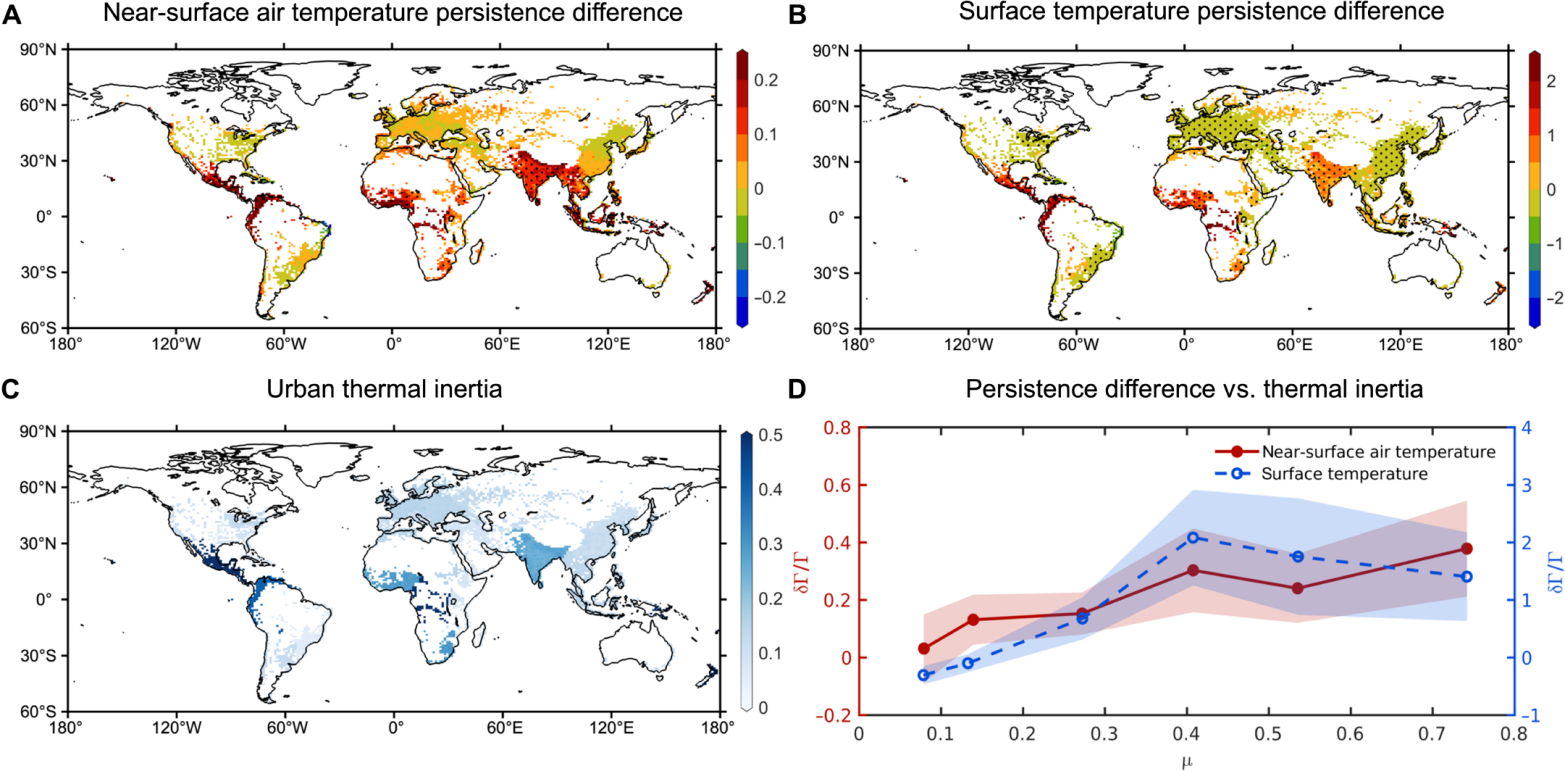
The urban-rural differences of near-surface air temperature and surface temperature persistence timescales and their relations with urban thermal inertia. (**A**) Urban-rural difference of near-surface air temperature persistence quantified by δΓ/Γ (the urban-rural difference in the persistence timescale normalized by the rural persistence timescale, where δ represents the urban-rural difference, namely, urban minus rural). The stippled regions are those with significant urban-rural differences of lag-1 autocorrelation at the 95% confidence level. (**B**) Urban-rural difference of surface temperature persistence quantified by δΓ/Γ. The stippled regions are those with significant urban-rural differences of lag-1 autocorrelation at the 95% confidence level. (**C**) Thermal inertia of urban impervious materials (μ, unit: 10^4^ J m^−2^ K^−1^ s^−1/2^) as used in the climate model. (**D**) Urban-rural differences of temperature persistence (δΓ/Γ) as a function of the thermal inertia of urban impervious materials (μ, unit: 10^4^ J m^−2^ K^−1^ s^−1/2^) in grid cells with significant urban-rural differences of lag-1 autocorrelation at the 95% confidence level. The large dots/circles in (D) are bin averages, and the shading indicates the SD in each bin.

To examine temperature persistence at local scales, urban and rural persistence timescales are estimated at the grid cell level. Before discussing the spatial pattern of urban-rural difference in the persistence timescale (Fig. 2), we highlight that most Γ values, for both urban and rural areas, range between 2 and 6 days (Fig. 1, C and F), which are commensurate with the timescale of synoptic weather variability. Furthermore, Fig. 1 (C and F) demonstrates the importance of Γ in the context of heat waves by showing that the average length of heat events, which are defined as periods when the daily temperature anomaly is positive and larger than 1 SD (*16*), is well correlated with Γ.

Red noise or, equivalently, a first-order autoregressive/Markov model is only an approximation for the full dynamics of daily temperature anomalies (*18*). Figure 1 shows that the autocorrelation does not experience a zero-crossing at large lags, indicating the existence of long-term memory in the temperature time series (*19*, *20*). However, at the timescales of interest (on the order of a few days), the red-noise approximation reasonably describes the autocorrelation and spectra (Fig. 1 and fig. S1), consistent with many meteorological studies on daily fluctuations (*21*, *22*). Further tests (see Materials and Methods) indicate that the primary findings of this study remain unaltered when using a decorrelation (or integral) timescale (*23*, *24*), which is free of the red-noise approximation.

### Urban-rural contrasts of temperature persistence

To quantify the local urban-rural difference in temperature persistence, the fractional difference of Γ between urban and rural land within the same grid cell (i.e., the urban-rural difference in Γ normalized by the rural Γ, or δΓ/Γ, where δ indicates the urban-rural difference) is computed (Fig. 2, A and B). Comparing Fig. 2A to Fig. 2B (see also Fig. 2D), it can be concluded that the urban-rural contrast of surface temperature persistence (Fig. 2B) is larger in terms of magnitude than its near-surface air temperature counterpart (Fig. 2A). This finding is expected as surface temperature is directly affected by surface radiative and biophysical properties. Any differences in surface radiative and biophysical properties between urban and rural land are reflected in the surface temperature. On the other hand, the mixing power of atmospheric turbulence tends to smear out or blend the effect of surface changes on the near-surface air temperature.

Globally, the urban-rural difference in temperature persistence can be either positive or negative. The negative δΓ/Γ values for near-surface air temperature (Fig. 2A) are mostly insignificant. In places where δΓ is significant for both near-surface air temperature (Fig. 2A) and surface temperature (Fig. 2B), urban near-surface air temperatures exhibit 10 to 40% higher persistence than their rural counterparts, and that number rises to 100 to 200% for urban surface temperatures. Central America, West Africa, and India experience the largest increases in urban near-surface air temperature persistence compared to their rural surroundings. This pattern is consistently observed across different seasons (fig. S2).

Why do Central America, West Africa, and India show the strongest urban-rural difference in terms of near-surface air temperature persistence? This is because the climate model prescribes large thermal inertia or thermal effusivity (μ, unit: 10^4^ J m^−2^ K^−1^ s^−1/2^) for urban impervious materials over these regions (Fig. 2C). The strong correlations between δΓ/Γ and thermal inertia of urban impervious materials are evidenced by Fig. 2D (coefficient of determination *R*^2^ = 0.86 and 0.63 for near-surface air temperature and surface temperature, respectively). In particular, the roofs in these tropical regions (Central America, West Africa, and India) are treated by the climate model as corrugated metal roofs with little or no insulation (*25*), which have exceptionally large thermal inertia (fig. S3). Known for their affordability, durability, weather resistance, ease of installation, and low maintenance, metal roofs are extensively used in tropical regions. Consequently, their impact on the urban-rural difference of temperature persistence is particularly evident in areas like Central America, West Africa, and India.

### A surface energy balance theory

The positive correlations between δΓ/Γ and the thermal inertia of urban impervious materials are physically intuitive and can be explained by a one-dimensional surface energy balance theory (see Materials and Methods). The surface energy balance model yields

$$\Gamma =\frac{\Omega }{1+\Omega }\Gamma_{f}$$

where Γ*_f_* is a forcing timescale and the nondimensional Ω parameter represents the importance of thermal inertia relative to energy dissipative mechanisms including sensible heat transfer, latent heat transfer, and radiative heat transfer. While the forcing timescale is identical for both urban and rural areas, the Ω parameter differs between urban and rural areas. Even without a priori defining the exact magnitude of Γ*_f_*, the surface energy balance theory predicts that as the thermal inertia of cities increases (e.g., using materials such as steel and dense concrete), the urban Ω parameter increases, leading to stronger urban surface temperature (and near-surface air temperature) persistence. This is in qualitative agreement with results shown in Fig. 2D.

Within the confines of the surface energy balance theory, the thickness of the urban impervious material that determines its extrinsic thermal mass plays a secondary role relative to that of the material’s intrinsic thermal inertia. This is also consistent with the climate model results. An examination of the relation between the climate model–simulated δΓ/Γ values and the thickness of impervious material prescribed by the climate model suggests that the simulated δΓ/Γ shows negative correlations with the thickness of roofs. This counterintuitive and unphysical result is because the corrugated metal roofs, which give rise to the large δΓ/Γ values, are relatively thin; this correlation is therefore not indicative of a causative link. Furthermore, the climate model–simulated δΓ/Γ shows no correlations with the thermal mass of roofs, which takes the material thickness into account. This finding shows that thermal inertia (rather than thermal mass) is the key control of daily temperature persistence.

The surface energy balance theory further provides a framework for analyzing how various environmental and meteorological factors such as urban morphology, vegetation fraction, wind speed, and air temperature might affect Γ (e.g., through altering energy dissipative mechanisms like sensible, latent, and radiative heat transfer). The fact that we focus on the role of thermal inertia in the analysis of climate model results does not imply that other factors do not play a role. However, since climate models inevitably have uncertainties in their inputs and parameterizations, comprehensively exploring the role of all possible factors affecting δΓ/Γ goes beyond the scope of this work. Nevertheless, two important points are highlighted here: (i) Some uncertainties in the climate model may be partially compensated for when analyzing differences between urban and rural areas within the same grid cell, and (ii) the linkage between larger thermal inertia of urban impervious materials and stronger urban surface (and near-surface air) temperature persistence, which is simulated by the climate model, can be elucidated through the surface energy balance analysis. The agreement between the climate model results and the surface energy balance theory provides credibility to the findings reported here.

### Further increases in urban near-surface air temperature persistence

How urban Γ will change if the thermal inertia of cities is further increased because of the continuing replacement of vegetation with impervious materials with large thermal inertia, such as steel and dense concrete, is now explored. The fractional increase in Γ or ΔΓ/Γ (the increase in Γ normalized by the baseline Γ) is computed. Here, Δ refers to a change over the urban land due to increased urbanization and is different from δ that indicates the urban-rural difference. From the surface energy balance theory, it can be shown that ΔΓ/Γ is independent of Γ*_f_* due to the normalization, increases with ΔΩ/Ω when ΔΩ/Ω is small (with stronger increasing trends in places with smaller Ω values), and approaches a constant when ΔΩ/Ω is large (see Materials and Methods).

**Fig. 3.**
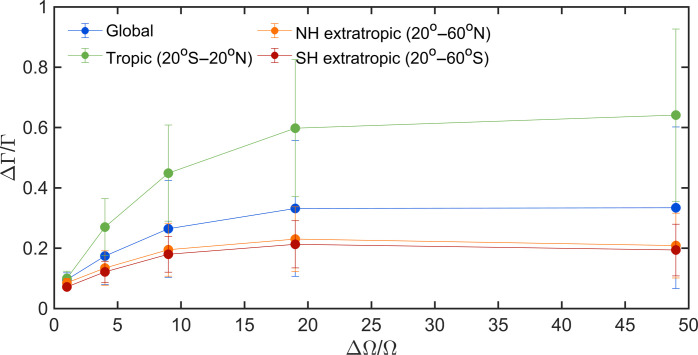
The relation between ΔΓ/Γ with ΔΩ/Ω in the uniform city simulations. NH and SH stand for the Northern Hemisphere and the Southern Hemisphere, respectively. Only grid cells with significant urban-rural differences of lag-1 autocorrelation at the 95% confidence level are considered. The large dots are bin averages, and the error bars indicate the SDs.

These behaviors are shown in Fig. 3, which depicts how further increases in thermal inertia of cities cause increases in Γ for the urban near-surface air temperature. In this analysis, cities are made of uniform building morphology and thermal properties globally to circumvent any uncertainties in the input data of these properties in the climate model, and the thermal inertia of impervious materials over urban land is gradually increased (see Materials and Methods). As predicted by the surface energy balance theory, the computed ΔΓ/Γ first increases with ΔΩ/Ω and then approaches a plateau. The increasing trend and concomitantly the plateau value are higher in the tropics (20 S to 20 N), compared to the extratropics (20° to 65°) in both Northern Hemisphere and Southern Hemisphere.

The fact that the increase of ΔΓ/Γ with ΔΩ/Ω is not spatially uniform in simulations with globally uniform buildings is an important result. It demonstrates that urban thermal inertia (or more broadly urban morphologies and properties) is not the only factor affecting ΔΓ/Γ, confirming an earlier point. Here, it is clear that the background climate also plays a role. The stronger increase of ΔΓ/Γ with ΔΩ/Ω in tropical regions implies that the baseline Ω values are smaller in the tropics than in other regions (Eq. 17). A plausible explanation is that the latent heat transfer efficiency is larger in the tropics with abundant rainfall (*26*), which leads to smaller baseline Ω values. This stronger increase of ΔΓ/Γ with ΔΩ/Ω in tropical regions also explains the significantly positive δΓ/Γ values in southeastern Asia in the control simulation (Fig. 2, A and B), although the thermal inertia of urban impervious materials in this region is not particularly large when compared to hotspots like Central America, West Africa, and India (Fig. 2C). Overall, these results suggest that the much stronger urban temperature persistence in the tropics compared to rural surroundings (Fig. 2, A and B) is not only related to the large thermal inertia of urban impervious materials prescribed by the input data (e.g., in regions like Central America, West Africa, and India) but also caused by the inherent stronger sensitivity of Γ to changes in thermal inertia in the tropics.

With a ninefold increase in Ω (e.g., from light wood to dense concrete or from asphalt to steel), Γ increases by 18 to 20% in the extratropical region (Fig. 3). To put these numbers in context, a linear relation between Γ and heat wave lengths as suggested by Fig. 1C would imply that heat wave lengths are also increased by 18 to 20%, which amounts to about 0.36 to 0.6 days since most heat waves are on the order of 2 to 3 days (*11*). This corresponds to an increase of mortality risk by 0.14 to 0.23% based on data for the United States (*11*). In the tropical region, the increase of mortality risk is even higher (0.34 to 0.51%) due to the much larger increase in Γ (45% for a ninefold increase of Ω; see Fig. 3). More worryingly, the longest heat waves will be extended the most.

## DISCUSSION

With the climatological UHIs (*6*) and potential synergistic interactions between heat waves and UHIs (*27*), it is already understood that urban temperatures will be much higher than rural temperatures during heat waves. Here, it is demonstrated that the large thermal inertia of urban impervious materials also leads to more persistent daily temperature anomalies in cities. Further increases in urban thermal inertia associated with continuing urbanization will cause even stronger urban temperature persistence. Cities in the tropical region are particularly prone to the effect of increasing thermal inertia when quantified by the fractional increase in the persistence timescale.

Urban heat mitigation often focuses on reducing the magnitude of temperature, especially the daytime maximum temperature. If so, it may appear plausible that increasing thermal inertia would reduce the daytime maximum temperature by storing more heat in the impervious materials (*3*–*5*). However, the consequence of increasing the thermal inertia is twofold: an increase in the nighttime minimum temperature (*3*–*5*) and an increase in the temperature persistence as shown here. The former effect of increasing thermal inertia at the diurnal scale has long been recognized (*6*), but the latter effect of increasing thermal inertia at the multiday timescale is the key finding offered here. Heat mitigation strategies that can simultaneously reduce the daytime maximum temperature and the thermal inertia should be considered. In light of these findings, replacing roofs that have exceptionally high thermal inertia such as noninsulated steel or dense concrete roofs with white roofs made of materials with lower thermal inertia is recommended.

Other solutions that may ameliorate urban temperature persistence include shading approaches (by trees or other means) that intercept solar radiation and release most of it rapidly into the air rather than storing it. A similar effect can be attained by solar panels but only if they are separated from the underlying roofs with an open-air space to reduce conductive storage. Roofs that enhance the latent heat transfer efficiency, such as blue and green roofs, are also possible but more costly alternatives. Water and wet soils also have large thermal inertia, and thus, blue and green roofs need to be properly designed to prevent any potential prolongation of temperature hazards. When achieving both goals (reducing the daytime maximum temperature and thermal inertia) is not possible, city planners need to quantify the benefits and penalties of proposed urban heat mitigation strategies not only at the diurnal timescale but also at longer (weather) timescales.

## MATERIALS AND METHODS

### Numerical simulations

The numerical experiments are conducted using the CESM version 2.0.1 (*28*). The land component of CESM2.0.1 is the Community Land Model (CLM) version 5 (*29*). Within each land grid cell, CLM allows for multiple land units including vegetated, crop, urban, glacier, and lake. The processes over the urban land unit are parameterized by the urban surface scheme of the CLM, called the CLM—Urban (CLMU). The CLMU conceptualizes the urban environment as a two-dimensional canyon that includes five facets (roof, sun-shaded wall, sun-lit wall, pervious ground, and impervious ground), of which four are impervious (roof, sun-shaded and sun-lit walls, and impervious ground). The CLMU parameterizes radiative, turbulent, and land surface processes within the canyon and aggregates the fluxes from different facets. Details about the CLMU model can be found elsewhere (*28*, *30*–*34*). The CLMU inputs, including building morphology and thermal properties, are supplied by a global dataset (*25*).

We use CESM to conduct global land-only (uncoupled from the atmospheric model) simulations. On the basis of an initial condition provided by CESM (that has been spun up), we run the CLM model at 0.9 latitude by 1.25 longitude for another 84 years of spin-up by recycling the 1990–2010 Global Soil Wetness Project Phase 3 atmospheric forcing (*29*) four times. We finally conduct multiple 21-year runs using the same atmospheric forcing from 1990 to 2010, initialized by the 84-year spin-up run, and our analysis focuses on the 20-year period from 1991 to 2010 from these final runs.

Two such sets of final simulations are conducted here. In the first set (called the control simulation), we do not alter any CLMU inputs but simply output all needed variables at the daily scale. The control simulation results are used for Figs. 1 and 2 and figs. S1 to S4. In the second set (called the uniform city simulations), we modify the CLMU input data so that all urban grid cells globally have the same building morphological and thermal properties representing those of the northeastern United States. Within the second set of simulations, we conduct a baseline run and five additional sensitivity runs with increased urban thermal inertia (by multiplying the heat capacity and thermal conductivity of all impervious facets by factors of 2, 5, 10, 20, and 50, respectively). ΔΓ/Γ and ΔΩ/Ω in Fig. 3 are computed as the differences between the sensitivity runs and the baseline run normalized by the results in the baseline run.

There are two reasons that the building morphological and thermal properties data are taken to be those representing the northeastern United States. First, the mortality numbers used here are from the United States, including the northeastern United States. Second, regardless of where these properties are taken from, the uniform city simulations are idealized simulations that are not meant to represent the real world but to test causal connections predicted by the surface energy balance theory. We expect that results from such simulations with other building properties agree with the theory.

#### Near-surface air temperature and surface temperature

Both urban and rural near-surface air temperatures (sometimes also termed surface air temperature or 2-m air temperature) are direct outputs from CLM. Within each grid cell where the urban fraction exceeds 0.1%, the urban near-surface air temperature is computed by CLMU for the urban land unit. For each land grid cell, the rural near-surface air temperature is an average for the vegetated and crop land units. The vegetated land unit might contain up to 15 different plant functional types and bare soil.

Urban and rural surface temperatures are inferred from the incoming longwave radiation (LW_in_, W m^−2^) and outgoing longwave radiation (LW_out_, W m^−2^) as $T_{s}={\left[\frac{{\mathrm{LW}}_{\text{out}}-\left(1-\varepsilon \right){\text{LW}}_{\text{in}}}{\varepsilon \sigma }\right]}^{1/4}$, where ε is the surface emissivity and σ is the Stefan-Boltzmann constant (5.67 × 10^−8^ W m^−2^ K^−4^). For consistency with prior work (*3*), the urban and rural surface emissivity values are set to be 0.88 and 0.96, respectively.

#### Autocorrelation

The autocorrelation (AC) is computed as

$$\text{AC}\left(\tau \right)=\frac{\bar{T\prime \left(t\right)T\prime \left(t+\tau \right)}}{\sigma_{T}^{2}}$$

where *T*′ is the daily temperature anomaly computed with the long-term linear trend and the mean annual cycle subtracted, the overbar denotes the time mean, τ denotes the time lag (unit: days), and $\sigma_{T}^{2}$ is the variance of *T*′. The mean annual cycle is the variation of temperature as a function of the day of the year but independent of the year and is computed as the average over 1991–2000. Sensitivity tests are conducted by only subtracting the mean annual cycle (but keeping the long-term linear trend), and the findings are not altered.

#### Spectrum

The spectrum is computed using Welch’s method with segments of length 2^9^ days and 50% overlap between different segments.

#### Spatial averaging

The spatially averaged AC values in Fig. 1 are calculated using Fisher *z* transformation. We transform AC values to their respective *z* values, compute the mean (and the 95% confidence intervals) of the *z* values, and then back-transform the mean (and the 95% confidence intervals) of the *z* values to obtain the mean (and the 95% confidence intervals) of the AC values, following prior work (*16*). The 95% confidence intervals are generally small because of the very large sample size.

### A surface energy balance theory for daily temperature persistence

The surface energy balance equation provides a theoretical basis for understanding how thermal inertia and various heat transfer mechanisms affect the persistence of surface temperature anomalies. It offers a direct physical connection between land surface properties and temperature persistence across different climatic zones.

The one-dimensional (or vertical) surface energy balance equation can be written as (*35*, *36*)

$${\text{SW}}_{\text{in}}\left(1-\alpha \right)+\varepsilon {\text{LW}}_{\text{in}}=H+\text{LE}+G+\varepsilon \sigma T_{s}^{4}$$ (1)

where SW_in_ and LW_in_ are the incoming shortwave and longwave radiation (W m^−2^), respectively; α and ε are the surface albedo and emissivity (unitless), respectively. The right-hand side of Eq. 1 includes four dissipative or energy loss terms (viewed from the surface perspective): the sensible heat flux (*H*, W m^−2^), the latent heat flux (LE, W m^−2^), the ground heat flux or heat storage (*G*, W m^−2^), and the emitted longwave radiation by the surface ($\varepsilon \sigma T_{s}^{4}$, W m^−2^), which is expressed using the Stefan-Boltzmann law where σ is the Stefan-Boltzmann constant (5.67 × 10^−8^ W m^−2^ K^−4^) and *T_s_* is the surface temperature (K). Here, the emitted longwave radiation is rearranged to the right-hand side of the surface energy balance equation, instead of the left-hand side as in textbooks (*35*, *36*). This reformulation emphasizes that each term on the right-hand side of Eq. 1 is a function of *T_s_*. In contrast, the terms on the left-hand side of Eq. 1 are assumed to be external (atmospheric) forcing onto the land surface and are not directly affected by *T_s_*.

To proceed, each term on the right-hand side of Eq. 1 is written as a linear function of *T_s_*. For example, a bulk parameterization is used for the sensible heat flux (*35*) given by

$$H=\frac{\rho c_{p}\left(T_{s}-T_{a}\right)}{r_{a}}$$ (2)

where ρ is the air density (kg m^−3^), *c_p_* is the heat capacity of dry air at constant pressure (J kg^−1^ K^−1^), *T_a_* is the air temperature (K), and *r_a_* is the aerodynamic resistance to convective heat transfer (s m^−1^) that depends on a range of factors such as the wind speed and thermal stratification of the near-surface atmosphere. This bulk parameterization for sensible heat flux can thus be written as follows

$$H=\lambda_{H}T_{s}+C_{H}$$ (3)

where λ*_H_* = ρ*c_p_*/*r_a_* and *C_H_* = −λ*_H_T_a_* represent the slope and the intercept of the linear relation between *H* and *T_s_*, respectively.

Likewise, for latent heat flux, the parameterization used is given by (*37*, *38*)

$$\text{LE}=\beta \frac{\rho L_{v}[q^{*}\left(T_{s}\right)-q_{a}]}{r_{a}}$$ (4)

where *L_v_* is the latent heat of vaporization (J kg^−1^), q^*^(*T_s_*) is the saturated specific humidity (kg kg^−1^) at the surface temperature and can be linked to the saturated water vapor pressure (Pa) at the surface temperature, or e^*^(*T_s_*), through q^*^(*T_s_*) = 0.622e^*^(*T_s_*)/*P*; *P* is the mean air pressure (Pa) at the surface; and *q_a_* is the air-specific humidity (kg kg^−1^) and similarly can be linked to the air water vapor pressure, or *e_a_*, through *q_a_* = 0.622*e_a_*/*P*. The β parameter is a dimensionless quantity (varying between 0 and 1) that reduces the actual evapotranspiration value from its potential value due to either dry soils or stressed vegetation. The latent heat flux as expressed in Eq. 4 is a function of *T_s_*. However, the relation is not linear since the saturated water vapor pressure (e^*^) is related to *T_s_* exponentially through the Clausius-Clapeyron relation. Nonetheless, the Clausius-Clapeyron relation can be linearized to yield

$$\text{LE}=\lambda_{\text{LE}}T_{s}+C_{\text{LE}}$$ (5)

where $\lambda_{\text{LE}}=\lambda_{H}\frac{\beta }{\gamma }{\text{∆}}_{a}$, $C_{\text{LE}}=\lambda_{\text{LE}}\left[-T_{a}+\frac{e^{*}\left(T_{a}\right)-e_{a}}{{\text{∆}}_{a}}\right]$, $\gamma =\frac{{\mathrm{Pc}}_{p}}{0.622L_{v}}$ is the psychometric constant, ${\text{∆}}_{a}={\frac{de^{*}}{\mathrm{dT}}|}_{T_{a}}$ is the derivative of e^*^ with respect to temperature evaluated at the air temperature, and e^*^(*T_a_*) is the saturated water vapor pressure at the air temperature. For ground heat flux, the force-restore model (*39*–*41*) is used, linking *G* to *T_s_* through

$$G=\frac{\mu }{\sqrt{2\omega }}\left[\frac{dT_{s}}{\mathrm{dt}}+\omega \left(T_{s}-T_{\text{deep}}\right)\right]$$ (6)

where μ is the thermal inertia or thermal effusivity (J m^−2^ K^−1^ s^−1/2^) and can be computed as $\mu =\sqrt{kC_{\mathrm{hp}}}$, where *k* is the thermal conductivity (J m^−1^ K^−1^ s^−1^) and *C_hp_* is the ground volumetric heat capacity (J m^−3^ K^−1^); ω is the angular frequency of the forcing [e.g., ω = 2π/(24 × 3600) = 7.27 × 10^−5^ rad s^−1^ for diurnal forcing and ω = 2π/365 = 0.0172 rad day^−1^ for annual forcing], and thus, ω^−1^ represents a forcing timescale; *T*_deep_ is the deep ground temperature taken at a depth that is unaffected by the thermal wave at the forcing timescale. The force-restore model was originally developed for soil/vegetated surfaces with stable *T*_deep_ at a sufficient depth. For building surfaces such as roofs and walls, application of the force-restore model assumes that energy storage over the timescale of interest is limited more severely by the thermal properties (volumetric heat capacity and thermal conductivity) than by the thickness and that the building interior temperature varies at a much slower rate than the surface temperature.

Here, the forcing timescale is assumed to be identical for both urban and rural areas. However, the exact magnitude of the forcing timescale is not essential for two reasons. First, the analysis focuses on the normalized persistence timescale difference (see, e.g., Figs. 2, A and B, and 3). Because of this normalization, the main findings are not affected by the exact magnitude of the forcing timescale. Second, the surface energy balance model is primarily used as a diagnostic tool (i.e., rather than a prognostic model) for interpreting the climate model results. Since this work analyzes daily temperature fluctuations, the forcing timescale must be longer than the diurnal scale (although its exact magnitude is not needed for our analysis). The focus on daily temperature fluctuations, instead of sub-daily temperature fluctuations, alleviates the complexity associated with considering multiple forcing timescales. For example, the diurnal forcing needs to be further included if some sub-daily temperatures (e.g., daily maximum or minimum temperature) were to be studied (*41*). Previous work found that the persistence of sub-daily temperatures differs from that of daily mean temperature (*42*). However, no theory that can adequately describe the persistence of sub-daily temperatures has been developed. Developing such a theory and exploring the persistence of sub-daily temperatures are left for future investigations.

Equation 6 can be rewritten as

$$G=\frac{\mu }{\sqrt{2\omega }}\left[\frac{dT_{s}}{\mathrm{dt}}+\omega \left(T_{s}-T_{\text{deep}}\right)\right]=\lambda_{G}\left(\Gamma_{f}\frac{dT_{s}}{\mathrm{dt}}\right)+\lambda_{G}T_{s}+C_{G}$$ (7)

where $\lambda_{G}=\mu \sqrt{\frac{\omega }{2}}$, Γ*_f_* = ω^−1^ (the forcing timescale), and *C_G_* = −λ*_G_T*_deep_.

The emitted longwave radiation involves the surface temperature to the fourth power, which can also be linearized to give

$$T_{s}^{4}\approx T_{a}^{4}+4T_{a}^{3}\left(T_{s}-T_{a}\right)$$ (8)

Hence, the emitted longwave radiation can be simplified to

$$\varepsilon \sigma T_{s}^{4}\approx \lambda_{\text{ELW}}T_{s}+C_{\text{ELW}}$$ (9)

where $\lambda_{\text{ELW}}=4\varepsilon \sigma T_{a}^{3}$, $C_{\text{ELW}}=-\frac{3}{4}\lambda_{\text{ELW}}T_{a}$.

Substituting Eqs. 3, 5, 7, and 9 into Eq. 1 yields

$$\frac{dT_{s}}{\mathrm{dt}}=-\frac{1}{\Gamma }T_{s}+C$$ (10)

where

$$\Gamma =\frac{\Omega }{1+\Omega }\Gamma_{f}$$ (11)

$$\Omega =\frac{\lambda_{G}}{\lambda_{H}+\lambda_{\text{LE}}+\lambda_{\text{ELW}}}=\frac{\lambda_{G}}{\lambda_{O}}$$ (12)

*C* = (λ*_G_*Γ*_f_*)^−1^[*SW_in_*(1 − α) + ε*LW_in_* − *C_H_* − *C*_LE_ − *C_G_* − *C*_ELW_]. Here Ω is a dimensionless parameter that quantifies the importance of thermal inertia (λ*_G_*) relative to three other heat dissipative mechanisms including sensible heat transfer, latent heat transfer, and radiative heat transfer (λ*_O_* = λ*_H_* + λ_LE_ + λ_ELW_).

The derivations so far largely follow a previous study (*38*) that examined the relative efficiencies of the four mechanisms on the right-hand side of Eq. 1 in dissipating surface temperature anomalies. However, the prior work (*38*) did not introduce the concept of temperature persistence within this surface energy balance framework, which is the primary focus of this study and is elaborated on in the next section.

#### The interpretation and estimation of Γ

When *T_s_* is decomposed into a mean component ($\bar{T_{s}}$) and a fluctuating component (${T\prime }_{s}$), it can be shown from Eq. 10 that

$$\frac{d{T\prime }_{s}}{\mathrm{dt}}=-\frac{1}{\Gamma }{T\prime }_{s}+C\prime$$ (13)

where *C*′ is the fluctuating component of *C*. Given the focus on the daily temperature persistence, the mean annual cycle is treated as the mean and the daily temperature anomalies are treated as the fluctuations. For the remaining derivation, we drop the prime symbol for notational convenience and state that *T_s_* is the temperature anomaly and *C* is the forcing anomaly.

If *C* is white noise, it can be shown that *T_s_* is red noise. This is essentially Hasselmann’s stochastic climate model (*12*, *13*), which has been widely used to study sea surface temperature anomalies (*43*, *44*). For a red noise process (also called a first-order autoregressive or Markov model), the stationary temporal autocorrelation is

$$\mathrm{AC}\left(\tau \right)=e^{-\frac{\tau }{\Gamma }}$$ (14)

where τ is the time lag (with a unit of days). It is clear that Γ characterizes the decay rate of the temporal autocorrelation, which explains why Γ is a good indicator for the daily temperature persistence. It can also be shown that the normalized spectrum of *T_s_* follows the Lorentz spectrum

$$\frac{E_{T}\left(f\right)}{\sigma_{T}^{2}}=\frac{4/\Gamma }{4\pi^{2}f^{2}+\Gamma^{-2}}$$ (15)

where *E_T_* is the energy spectrum of $T_{s}$, $\sigma_{T}^{2}$ is the variance of *T_s_*, and *f* is the frequency (with a unit of day^−1^). Equation 15 indicates that the normalized *T_s_* spectrum approaches *f*^−2^ at high frequencies (red noise) and *f*^0^ at low frequencies (white noise). The transition of the two scaling laws occurs at a frequency of 1/(2πΓ), corresponding to a timescale of ~Γ. Again, this demonstrates that Γ is an important timescale characterizing the dynamics of *T_s_*. An alternative way of identifying the transitional frequency (or the transitional timescale) is to use the normalized, premultiplied spectrum ${\mathrm{fE}}_{T}\left(f\right)/\sigma_{T}^{2}$, whose peak is reached at the transitional frequency.

Hence, Γ can be estimated from lag-1 autocorrelation AC(1) as

$$\Gamma ={\left\{-\text{ln}\left[\text{AC}\left(1\right)\right]\right\}}^{-1}$$ (16)

We could also estimate Γ using the premultiplied spectrum, but identifying the peak of the premultiplied spectrum can be ambiguous. We choose to use the decay of the autocorrelation to estimate Γ for its simplicity and following recent studies (*16*, *43*). In the analysis of numerical simulation results, a significant urban-rural difference in Γ indicates that the difference in AC(1) between urban and rural areas is significant at the 95% confidence level. This significance is determined using a two-tailed test, which uses *z* scores derived from the Fisher transformation.

#### Changes in Γ due to further increases in urban thermal inertia

From Eq. 11, changes in Ω (represented by ΔΩ) over urban land will cause a change in Γ (represented by ΔΓ). We are particularly interested in the fractional change in Γ due to fractional changes in Ω. When ΔΩ is sufficiently small

$$\frac{\Delta \Gamma }{\Gamma }=\left(\frac{1}{1+\Omega }\right)\frac{\Delta \Omega }{\Omega }$$ (17)

This expression states that ΔΓ/Γ increases with ΔΩ/Ω when ΔΩ/Ω is small and the increasing trend is stronger in places with smaller Ω. On the other hand, Eq. 11 indicates that as Ω → ∞, Γ → Γ_f_. In other words, when Ω is sufficiently large, any additional increase in Ω will no longer cause Γ to increase. This is understandable as when the thermal inertia is large enough, any further increase in thermal inertia will no longer cause temperature persistence to increase.

On the basis of Eq. 12, fractional changes in Ω can be further linked to fractional changes in thermal inertia through

$$\frac{\Delta \Omega }{\Omega }=\frac{\Delta \lambda_{G}}{\lambda_{G}}-\frac{\Delta \lambda_{O}}{\lambda_{O}}\approx \frac{\Delta \lambda_{G}}{\lambda_{G}}=\frac{\Delta \mu }{\mu }$$ (18)

Here, we have implicitly assumed that increases in thermal inertia will not cause λ_O_ to change strongly because λ_O_ is primarily controlled by sensible, latent, and radiative heat transfer efficiencies that are, to a leading order, not sensitive to changes in thermal inertia.

#### The surface energy balance model as a diagnostic tool

The correspondence and difference between the surface temperature in the surface energy balance model and the surface temperature simulated by the climate model requires further clarification. The surface energy balance model is a one-dimensional model for a homogeneous surface, and thus, the surface temperature represents a bulk temperature. In contrast, the surface temperature simulated by the climate model is an “averaged” or “aggregated” surface temperature of multiple surfaces/facets since the climate model considers the energy balances for multiple surfaces/facets for both urban and rural land. As a result, the surface energy balance model can be viewed as a simplified version of the climate model and a diagnostic tool for understanding the persistence of surface temperature simulated by the more complicated climate model (*45*).

The air temperature in the surface energy balance model is also different from the near-surface air temperature simulated by the climate model. The air temperature in the surface energy balance model is a forcing for the bulk surface and thus better corresponds to the atmospheric forcing temperature used to drive the climate model instead of the simulated near-surface air temperature. In contrast, the near-surface air temperature simulated by the climate model can be viewed as an interpolated temperature between the surface temperature and the atmospheric forcing temperature (either following the well-established Monin-Obukhov similarity theory or some empirical parameterizations or a combination of both). Hence, with the atmospheric forcing anomalies viewed as white noise, the day-to-day variability of near-surface air temperature simulated by the climate model largely follows the day-to-day variability of surface temperature simulated by the climate model, but damped by the mixing power of atmospheric turbulence (*45*). This provides the theoretical basis for using the surface energy balance model to qualitatively diagnose the persistence of near-surface air temperature simulated by the climate model.

### A decorrelation timescale that does not invoke the red-noise approximation

A decorrelation (or integral) timescale can be defined without a priori assuming that temperature anomalies follow a red-noise process, as follows (*23*, *24*)

$$T_{o}=1+2\sum_{L=1}^{N}\left(1-\frac{L}{N}\right)\text{AC}\left(L\right)$$ (19)

where *L* is the lag ranging from 1 to *N* and AC(*L*) is the autocorrelation at lag *L*. Unlike the Γ estimated from lag-1 autocorrelation, which indicates short-term memory, the decorrelation timescale *T*_o_ encodes all the information about the autocorrelation, including long-term memory effects (*14*).

Figure S4 shows the results for δ*T*_o_/*T*_o_ computed with *N* = 90. The broad pattern of δ*T*_o_/*T*_o_ remains similar to δΓ/Γ, although *T*_o_ and Γ are different. Hence, the main conclusions here are not affected by the red-noise approximation. Note that for continuous time series with *N* → ∞, $T_{o}=1+2\int_{0}^{\infty }\text{AC}\left(\tau \right)d\tau$. For the specific choice of an exponential autocorrelation function usually associated with a first-order autoregressive process, *T*_o_ = 1 + 2Γ.
