# Supplementary material for: Persistent urban heat
Source: Sci Adv. 2024 Apr 10;10(15):eadj7398. doi: 10.1126/sciadv.adj7398 (PMC11006209; doi:10.1126/sciadv.adj7398)
Supplement: Supplementary file 1 — Figs. S1 to S4 [file sciadv.adj7398_sm.pdf]

Science Advances
AAAS

Supplementary Materials for

Persistent urban heat

Dan Li *et al.*

Corresponding author: Dan Li, lidan@bu.edu

Sci. Adv. **10**, eadj7398 (2024)
DOI: 10.1126/sciadv.adj7398

This PDF file includes:

Figs. S1 to S4

Supplementary Materials

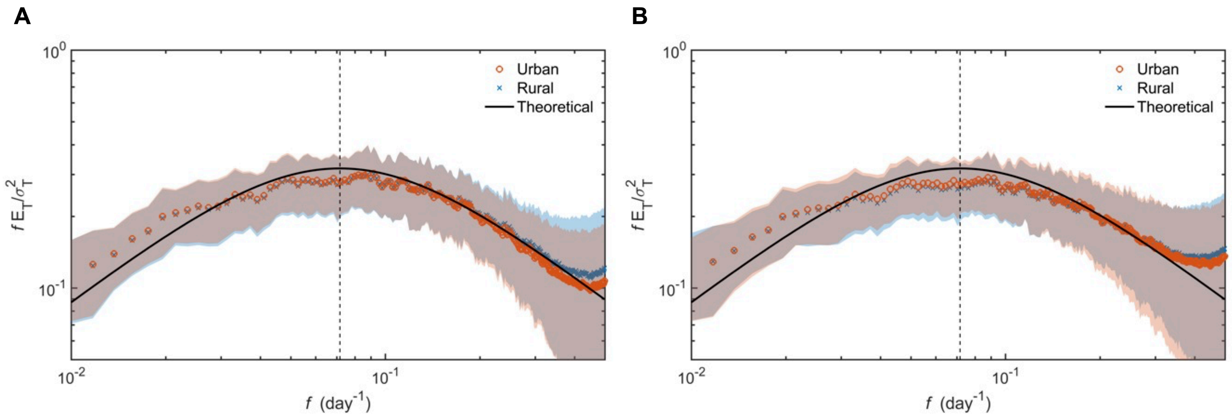

Fig. S1. The pre-multiplied spectra of urban and rural temperature anomalies normalized by their variances as a function of frequency (*f*). (A) Near-surface air temperature. (B) Surface temperature. The black line is the theoretical result for a red-noise process with a transitional frequency indicated by the vertical dashed line of $1/(2\pi\Gamma)$. The spectra are averaged over all grid cells that have urban fractions larger than 0.1% and the shading indicate standard deviations.

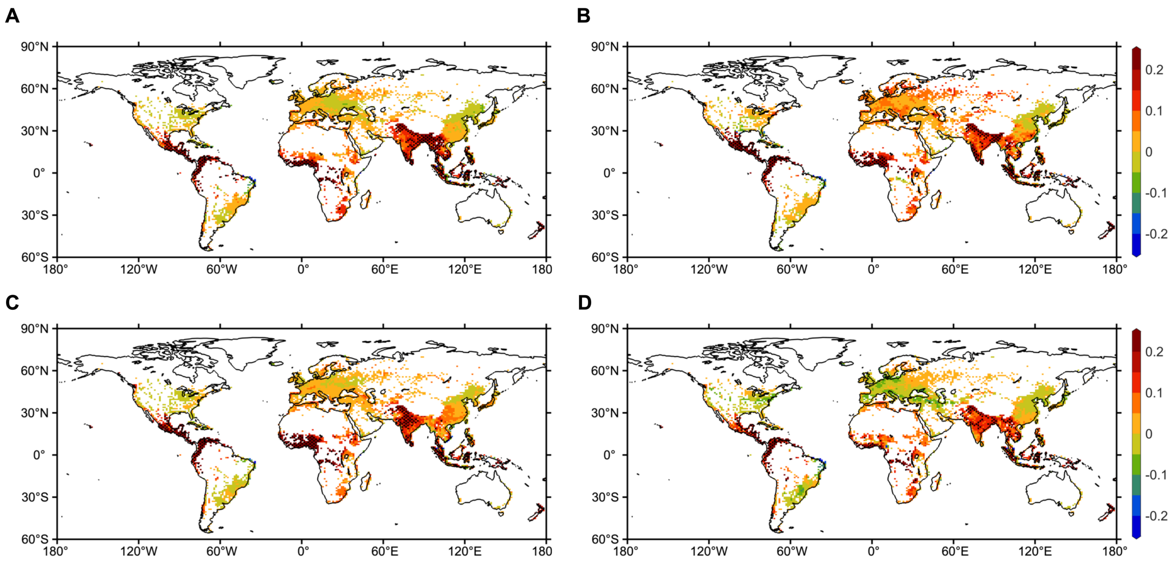

Fig. S2. The urban-rural differences of near-surface air temperature persistence in different seasons. (A) Spring; (B) Summer; (C) Autumn; (D) Winter. The stippled regions are those with significant urban-rural differences of lag-1 autocorrelation.

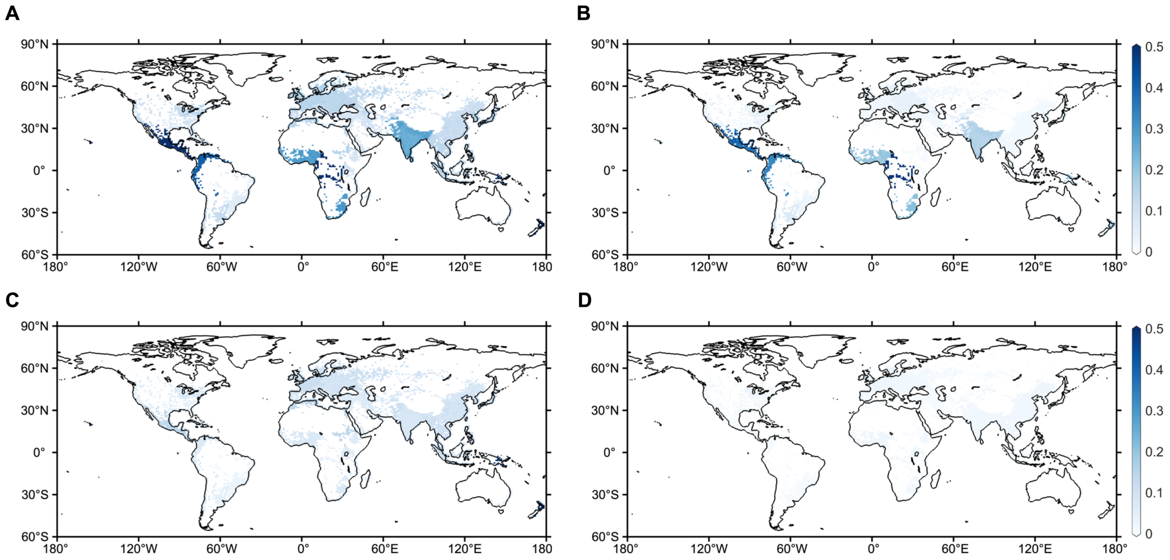

Fig. S3. Thermal inertia of urban impervious materials (μ, unit: 10^4 J m^{-2} K^{-1} $s^{-1/2}$). (A) Total, (B) Roof, (C) Wall, and (D) Impervious ground.

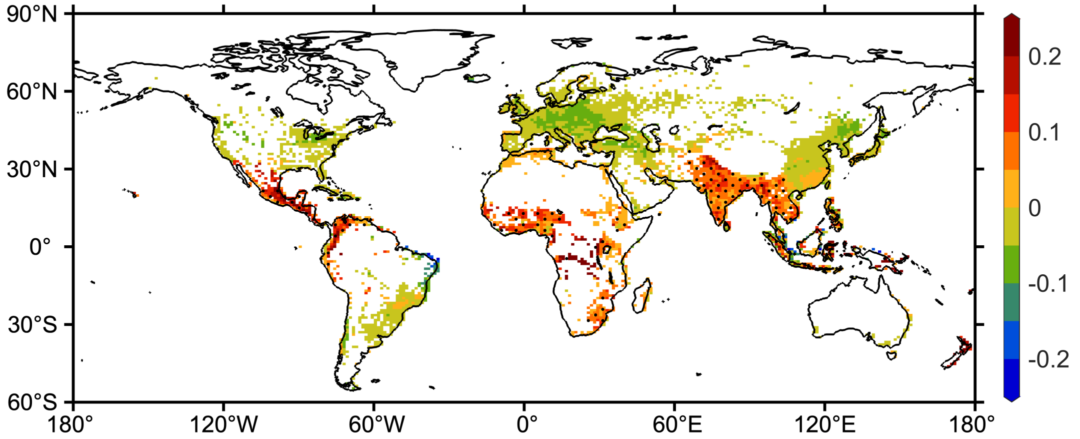

Fig. S4. The urban-rural difference of near-surface air temperature decorrelation time scale. The stippled regions are those with significant urban-rural differences of lag-1 autocorrelation.
